# Supplementary material for: Factors associated with pneumococcal carriage and density in children and adults in Fiji, using four cross-sectional surveys
Source: PLoS One. 2020 Apr 1;15(4):e0231041. doi: 10.1371/journal.pone.0231041 (PMC7112956; doi:10.1371/journal.pone.0231041)
Supplement: S1 Table — (DOCX) [file pone.0231041.s001.docx]

**S1 Table: Unadjusted and adjusted odds ratios of non-PCV10 pneumococcal carriage in association with participant characteristics in four cross-sectional carriage surveys pre-PCV10 (2012) and annually thereafter (2013–2015) in Fiji (n = 7,962).**

| **Exposure** | | **Non-PCV10 carriage^a^**  **n / N (%)** | **Unadjusted odds ratio (95% CI)** | ***P*-value** | **Adjusted odds ratio (95% CI)** | ***P*-value** |
| --- | --- | --- | --- | --- | --- | --- |
| **PCV10 vaccination status** | |  |  | <0.001 |  | 0.856 |
|  | Not vaccinated | 1548 / 6875 (22.5) | *ref* |  | *ref* |  |
|  | Vaccinated^b^ | 357 / 1087 (32.8) | 1.68 (1.46 – 1.93) |  | 1.02 (0.82 – 1.28) |  |
| **Survey year** | |  |  | <0.001 |  | 0.080 |
|  | Pre-PCV10 (2012) | 489 / 1975 (24.8) | *ref* |  | *ref* |  |
|  | 1 year post-PCV10 (2013) | 508 / 2022 (25.1) | 1.02 (0.88 – 1.18) |  | 0.87 (0.64 – 1.19) |  |
|  | 2 years post-PCV10 (2014) | 352 / 1987 (17.7) | 0.65 (0.56 – 0.76) |  | 0.65 (0.46 – 0.91) |  |
|  | 3 years post-PCV10 (2015) | 556 / 1978 (28.1) | 1.19 (1.03 – 1.37) |  | 0.75 (0.53 – 1.05) |  |
| **Ethnicity** | |  |  | <0.001 |  | <0.001 |
|  | Fijian of Indian Descent | 348 / 3206 (10.9) | *ref* |  | *ref* |  |
|  | iTaukei | 1557 / 4756 (32.7) | 4.00 (3.52 – 4.54) |  | 2.71 (2.08 – 3.52) |  |
| **Participant group** | |  |  | <0.001 |  | <0.001 |
|  | Caregivers | 153 / 2029 (7.5) | *ref* |  | *ref* |  |
|  | Infants (5 – 8 weeks) | 393 / 1946 (20.1) | 3.10 (2.54 – 3.78) |  | 3.62 (2.91 – 4.50) |  |
|  | Toddlers (12 – 23 months) | 642 / 1972 (32.6) | 5.92 (4.90 – 7.16) |  | 6.23 (4.91 – 7.89) |  |
|  | Children (2 – 6 years) | 717 / 2015 (35.6) | 6.77 (5.61 – 8.18) |  | 7.14 (5.80 – 8.79) |  |
| **Residential location** | |  |  | <0.001 |  | <0.001 |
|  | Rural | 849 / 3880 (21.9) | *ref* |  | *ref* |  |
|  | Urban | 1056 / 4082 (25.9) | 1.25 (1.12 – 1.38) |  | 1.30 (1.16 – 1.46) |  |
| **Participant sex** | |  |  | <0.001 |  | 0.635 |
|  | Male | 918 / 3358 (27.3) | *ref* |  | *ref* |  |
|  | Female | 987 / 4604 (21.4) | 0.73 (0.65 – 0.80) |  | 1.03 (0.91 – 1.16) |  |
| **Number of children < 5 years living in the household^c^** | |  |  | <0.001 |  | <0.001 |
|  | Less than two | 714 / 4035 (17.7) | *ref* |  | *ref* |  |
|  | Two or more | 1191 / 3924 (30.4) | 2.03 (1.82 – 2.25) |  | 1.47 (1.30 – 1.65) |  |
| **Family income level^d^** | |  |  | <0.001 |  | <0.001 |
|  | Not low | 640 / 3175 (20.2) | *ref* |  | *ref* |  |
|  | Low | 1174 / 4518 (26.0) | 1.39 (1.25 – 1.55) |  | 1.38 (1.22 – 1.56) |  |
| **Symptoms of URTI** | |  |  | <0.001 |  | <0.001 |
|  | Not present | 1178 / 5903 (20.0) | *ref* |  | *ref* |  |
|  | Present | 727 / 2059 (35.5) | 2.19 (1.96 – 2.45) |  | 1.64 (1.45 – 1.87) |  |
| **Household cigarette smoke** | |  |  | 0.575 |  |  |
|  | No exposure | 872 / 3689 (23.6) | *ref* |  |  |  |
|  | Exposure | 1033 / 4273 (24.2) | 1.03 (0.93 – 1.14) |  |  |  |
| **Antibiotic use in previous fortnight^e^** | |  |  | 0.771 |  |  |
|  | Not used | 1823 / 7610 (24.0) | *ref* |  |  |  |
|  | Used | 81 / 348 (23.3) | 0.96 (0.75 – 1.24) |  |  |  |

Abbreviations: CI, confidence interval; PCV10, ten-valent pneumococcal conjugate vaccine; URTI, upper respiratory tract infection. ^a^Pneumococcal serotypes not included in PCV10, including non-encapsulated lineages; ^b^Two doses of PCV10 given before the age of 12 months, or one or more doses of PCV10 given at or after 12 months of age [34]; ^c^ Data on number of children under five years living in the household were missing for three participants, of whom none were non-PCV10 pneumococcal carriers; ^d^Weekly family income below the basic needs poverty line (<FJ$175 per week)[28]; data on family income were was missing for 269 participants, of whom 91 were non-PCV10 pneumococcal carriers; ^e^Data on antibiotics use were missing for four participants, of whom one was a non-PCV10 pneumococcal carrier.
