# Supplementary material for: Factors associated with pneumococcal carriage and density in children and adults in Fiji, using four cross-sectional surveys
Source: PLoS One. 2020 Apr 1;15(4):e0231041. doi: 10.1371/journal.pone.0231041 (PMC7112956; doi:10.1371/journal.pone.0231041)
Supplement: S2 Table — (DOCX) [file pone.0231041.s002.docx]

**S2 Table: Unadjusted and adjusted differences in medians of PCV10 pneumococcal carriage density in association with participant characteristics in four cross-sectional carriage surveys pre-PCV10 (2012) and annually thereafter (2013–2015) in Fiji (n = 708).**

| **Exposure** | | **Density of vaccine-type pneumococcal carriage^a^**  **(log10 GE/ml)**  **n, median / IQR (%)** | **Unadjusted**  **median difference**  **(95% CI)** | ***P*-value** | **Adjusted**  **median difference**  **(95% CI)** | ***P*-value** |
| --- | --- | --- | --- | --- | --- | --- |
| **PCV10 vaccination status** | |  |  | 0.022 |  | 0.258 |
|  | Not vaccinated | 629, 5.0 (4.1 – 5.6) | *ref* |  | *ref* |  |
|  | Vaccinated^b^ | 79, 4.5 (3.6 – 5.5) | -0.42 (-0.78, -0.06) |  | -0.25 (-0.67, 0.18) |  |
| **Survey year** | |  |  | 0.002 |  | 0.015 |
|  | Pre-PCV10 (2012) | 275, 4.9 (4.1 – 5.6) | *ref* |  | *ref* |  |
|  | 1 year post-PCV10 (2013) | 216, 5.2 (4.3 – 5.7) | 0.28 (0.02, 0.54) |  | 0.32 (0.08, 0.56) |  |
|  | 2 years post-PCV10 (2014) | 102, 4.8 (4.0 – 5.7) | -0.12 (-0.45, 0.22) |  | -0.09 (-0.43, 0.25) |  |
|  | 3 years post-PCV10 (2015) | 115, 4.5 (3.8 – 5.3) | -0.34 (-0.66, -0.02) |  | -0.16 (-0.50, 0.18) |  |
| **Ethnicity** | |  |  | 0.968 |  | 0.810 |
|  | Fijian of Indian Descent | 147, 4.9 (4.1 – 5.7) | *ref* |  | *ref* |  |
|  | iTaukei | 561, 4.9 (4.1 – 5.6) | 0.01 (-0.27, 0.28) |  | -0.03 (-0.22, 0.28) |  |
| **Participant group** | |  |  | 0.431 |  | 0.437 |
|  | Caregivers | 41, 4.6, (3.9 – 5.3) | *ref* |  | *ref* |  |
|  | Infants (5 – 8 weeks) | 121, 5.0 (4.2 – 5.7) | 0.37 (-0.17, 0.91) |  | 0.24 (-0.24, 0.71) |  |
|  | Toddlers (12 – 23 months) | 277, 5.0 (4.2 – 5.7) | 0.36 (-0.14, 0.86) |  | 0.31 (-0.14, 0.77) |  |
|  | Children (2 – 6 years) | 269, 4.8 (4.0 – 5.6) | 0.23 (-0.27, 0.73) |  | 0.15 (-0.29, 0.59) |  |
| **Residential location** | |  |  | 0.701 |  |  |
|  | Rural | 304, 4.9 (4.0 – 5.7) | *ref* |  |  |  |
|  | Urban | 404, 4.9 (4.2 – 5.6) | 0.05 (-0.19, 0.28) |  |  |  |
| **Participant sex** | |  |  | 0.963 |  |  |
|  | Male | 352, 4.9 (4.0 – 5.6) | *ref* |  |  |  |
|  | Female | 356, 4.9 (4.1 – 5.6) | -0.01 (-0.22, 0.24) |  |  |  |
| **Number of children < 5 years living in the household** | |  |  | 0.089 |  | 0.078 |
|  | Less than two | 283, 5.0 (4.2 – 5.7) | *ref* |  |  |  |
|  | Two or more | 425, 4.8 (4.0 – 5.6) | -0.19 (-0.42, 0.03) |  | -0.18 (-0.39, 0.02) |  |
| **Family income level^c^** | |  |  | 0.369 |  |  |
|  | Not low | 207, 4.8 (4.1 – 5.6) | *ref* |  |  |  |
|  | Low | 463, 5.0 (4.0 – 5.7) | 0.11 (-0.13, 0.36) |  |  |  |
| **Symptoms of URTI** | |  |  | 0.038 |  | 0.002 |
|  | Not present | 435, 4.8 (4.0 – 5.6) | *ref* |  | *ref* |  |
|  | Present | 273, 5.0 (4.2 – 5.8) | 0.24 (0.01, 0.47) |  | 0.32 (0.11, 0.53) |  |
| **Household cigarette smoke** | |  |  | 0.405 |  |  |
|  | No exposure | 283, 5.0 (4.1 – 5.6) | *ref* |  |  |  |
|  | Exposure | 425, 4.9 (4.0 – 5.6) | -0.10 (-0.32, 0.13) |  |  |  |
| **Antibiotic use in previous fortnight^d^** | |  |  | 0.385 |  |  |
|  | Not used | 663, 4.9 (4.1 – 5.6) | *ref* |  |  |  |
|  | Used | 44, 5.1 (4.3 – 5.6) | 0.20 (-0.25, 0.65) |  |  |  |

Abbreviations: CI, confidence interval; PCV10, ten-valent pneumococcal conjugate vaccine; URTI, upper respiratory tract infection. ^a^Density of pneumococcal serotypes included in PCV10 (serotypes 1, 4, 5, 6B, 7F, 9V, 14, 18C, 19F, and 23F); ^b^Two doses of PCV10 given before the age of 12 months, or one or more doses of PCV10 given after 12 months of age[34]; ^c^ Weekly family income below the basic needs poverty line (<FJ$175 per week)[28]; data on family income were missing for 38 PCV10 pneumococcal carriers**; ^d^** Data on antibiotic use was missing for one PCV10 pneumococcal carrier.
