# Supplementary material for: Factors associated with pneumococcal carriage and density in children and adults in Fiji, using four cross-sectional surveys
Source: PLoS One. 2020 Apr 1;15(4):e0231041. doi: 10.1371/journal.pone.0231041 (PMC7112956; doi:10.1371/journal.pone.0231041)
Supplement: S3 Table — (DOCX) [file pone.0231041.s003.docx]

**S3 Table: Unadjusted and adjusted differences in medians of non-PCV10 pneumococcal carriage density in association with participant characteristics in four cross-sectional carriage surveys pre-PCV10 (2012) and annually thereafter (2013–2015) in Fiji (n = 1,905).**

| **Exposure** | | **Density of non-vaccine-type pneumococcal carriage^a^**  **(log10 GE/ml)**  **n, median / IQR (%)** | **Unadjusted**  **median difference**  **(95% CI)** | ***P*-value** | **Adjusted**  **median difference**  **(95% CI)** | ***P*-value** |
| --- | --- | --- | --- | --- | --- | --- |
| **PCV10 vaccination status** | |  |  | 0.968 |  | 0.225 |
|  | Not vaccinated | 1548, 4.9 (4.1 – 5.7) | *ref* |  | *ref* |  |
|  | Vaccinated^b^ | 357, 4.9 (4.0 – 5.7) | 0.00 (-0.17, 0.17) |  | -0.15, (-0.40, 0.10) |  |
| **Survey year** | |  |  | 0.022 |  | <0.001 |
|  | Pre-PCV10 (2012) | 489, 4.8 (4.0 – 5.6) | *ref* |  | *ref* |  |
|  | 1 year post-PCV10 (2013) | 508, 5.0 (4.2 – 5.9) | 0.24 (0.06, 0.42) |  | 0.34 (0.16, 0.53) |  |
|  | 2 years post-PCV10 (2014) | 352, 5.0 (4.1 – 5.8) | 0.22 (0.01, 0.42) |  | 0.33 (0.11, 0.55) |  |
|  | 3 years post-PCV10 (2015) | 556, 4.8 (4.0 – 5.7) | 0.04 (-0.13, 0.22) |  | 0.12 (-0.08, 0.33) |  |
| **Ethnicity** | |  |  | 0.147 |  | 0.196 |
|  | Fijian of Indian Descent | 348, 4.8 (4.0 – 5.7) | *ref* |  | *ref* |  |
|  | iTaukei | 1557, 4.9 (4.1 – 5.7) | 0.13 (-0.04, 0.30) |  | 0.11 (-0.06, 0.29) |  |
| **Participant group** | |  |  | 0.033 |  | 0.010 |
|  | Caregivers | 153, 4.6 (3.9 – 5.4) | *ref* |  | *ref* |  |
|  | Infants (5 – 8 weeks) | 393, 4.8 (4.1 – 5.7) | 0.17 (-0.10, 0.44) |  | 0.31 (0.04, 0.59) |  |
|  | Toddlers (12 – 23 months) | 642, 4.9 (4.0 – 5.7) | 0.26 (0.01, 0.52) |  | 0.41 (0.13, 0.70) |  |
|  | Children (2 – 6 years) | 717, 5.0 (4.2 – 5.8) | 0.34 (0.09, 0.59) |  | 0.43 (0.17, 0.69) |  |
| **Residential location** | |  |  | 0.479 |  |  |
|  | Rural | 849, 4.9 (4.0 – 5.7) | *ref* |  |  |  |
|  | Urban | 1056, 4.9 (4.1 – 5.7) | 0.05 (-0.10, 0.18) |  |  |  |
| **Participant sex** | |  |  | 0.663 |  |  |
|  | Male | 918, 4.9 (4.1 – 5.7) | *ref* |  |  |  |
|  | Female | 987, 4.9 (4.1 – 5.8) | 0.03 (-0.10, 0.16) |  |  |  |
| **Number of children < 5 years living in the household** | |  |  | 0.766 |  |  |
|  | Less than two | 714, 4.9 (4.1 – 5.8) | *ref* |  |  |  |
|  | Two or more | 1191, 4.9 (4.1 – 5.7) | -0.02 (-0.16, 0.11) |  |  |  |
| **Family income level^c^** | |  |  | 0.391 |  |  |
|  | Not low | 640, 4.9 (4.1 – 5.7) | *ref* |  |  |  |
|  | Low | 1174, 4.9 (4.1 – 5.7) | -0.06 (-0.20, 0.08) |  |  |  |
| **Symptoms of URTI** | |  |  | 0.002 |  | <0.001 |
|  | Not present | 1178, 4.8 (4.0 – 5.6) | *ref* |  | *ref* |  |
|  | Present | 727, 5.1 (4.2 – 5.9) | 0.24 (0.09, 0.38) |  | 0.29 (0.15, 0.43) |  |
| **Household cigarette smoke** | |  |  | 0.831 |  |  |
|  | No exposure | 872, 4.9 (4.1 – 5.7) | *ref* |  |  |  |
|  | Exposure | 1033, 4.9 (4.1 – 5.8) | 0.01 (-0.12, 0.15) |  |  |  |
| **Antibiotic use in previous fortnight^d^** | |  |  | 0.269 |  |  |
|  | Not used | 1823, 4.9 (4.1 – 5.7) | *ref* |  |  |  |
|  | Used | 81, 5.1 (4.4 – 5.7) | 0.19 (-0.15, 0.53) |  |  |  |

Abbreviations: CI, confidence interval; PCV10, ten-valent pneumococcal conjugate vaccine; URTI, upper respiratory tract infection. ^a^ Density of pneumococcal serotypes not included in PCV10, including non-encapsulated pneumococci; ^b^Two doses of PCV10 given before the age of 12 months, or one or more doses of PCV10 given at or after 12 months of age[34]; ^c^ Weekly family income below the basic needs poverty line (<FJ$175 per week)[28]; data on family income were missing for 91 non-PCV10 pneumococcal carriers; **^d^** Data on antibiotic use were missing for two non-PCV10 pneumococcal carriers.
